# Blood Product Transfusion and Coagulopathy in Children with Traumatic Brain Injury: A Narrative Review

**DOI:** 10.3390/children13010104

**Published:** 2026-01-11

**Authors:** Robert Marcel T. Huibonhoa, Niranjan Vijayakumar, Daniel Kelly, Oliver Karam, Madhuradhar Chegondi

**Affiliations:** 1 Department of Pediatrics, Division of Critical Care, Children’s Hospital of Illinois at OSF Healthcare, University of Illinois College of Medicine, Peoria, IL 61605, USA; chegondi@uic.edu; 2 Department of Pediatrics, Division of Pediatric Critical Care Medicine, Children’s Mercy Hospital, University of Missouri-Kansas City, Kansas City, MO 64108, USA; nvijayakumar1@cmh.edu; 3 Department of Pediatrics, Division of Medical Critical Care, Boston Children’s Hospital, Harvard Medical School, Boston, MA 02115, USA; 4 Department of Pediatrics, Section of Critical Care, Yale School of Medicine, New Haven, CT 06520, USA

**Keywords:** traumatic brain injury, pediatric, blood transfusion

## Abstract

Traumatic brain injury (TBI) is a leading cause of critical illness and mortality in children. Transfusion of blood products, a common intervention in the management of pediatric TBI, has important implications for related principles, including trauma-induced coagulopathy, cerebral perfusion, and cerebral oxygen delivery. Knowledge gaps persist due to the limited availability of pediatric-specific data regarding blood product transfusion in TBI. In particular, there is a lack of prospective studies defining appropriate specific laboratory thresholds and transfusion targets, as well as insufficient evidence to guide the weighing of potential benefits against transfusion-related risks in this population. Although blood product transfusion in pediatric TBI has been associated with worse clinical outcomes, the underlying mechanisms and contributing factors remain poorly understood. In this review, we aimed to describe the pediatric literature on component and whole blood product transfusion in children with TBI and the pathophysiological mechanisms underlying the development of coagulopathy in this population. In addition, we incorporated available pediatric guidelines and recommendations specific to the setting of acute brain injury.

## 1. Introduction

Traumatic brain injury (TBI) is a leading cause of critical illness in children [1]. The estimated annual incidence of pediatric TBI is between 47 and 280 cases per 100,000 population globally, with a bimodal age distribution of less than 3 years and 15 to 18 years of age [2]. TBI, along with hemorrhagic shock and its sequelae, is the primary driver of mortality in injured children [3]. Children with TBI are more likely to receive blood product transfusions due to their association with other injuries leading to hemorrhagic circulatory collapse requiring volume resuscitation, and/or the presence of coagulopathy. Trauma-induced coagulopathy (TIC) is an entity heavily debated upon, but typically defined by the presence of impaired coagulation parameters such as a prolonged prothrombin time (PT), prolonged activated partial thromboplastin time (aPTT), or increased International Normalized Ratio (INR), leading to hemostatic, immune, and inflammatory changes in the setting of trauma [4,5,6,7]. Although TIC is commonly studied in adults, its understanding in children is limited and has not been well explored [7]. In the setting of isolated TBI, coagulopathy also occurs, particularly in severe TBI (sTBI), and may involve mechanisms distinct from TIC [8,9]. TBI-associated coagulopathy is a recognized risk factor for secondary brain injury and is independently associated with adverse outcomes [10]. Even in isolated TBI without other associated injuries, conflict exists in balancing the correction of laboratory derangements indicative of coagulopathy with the need for placement of invasive intracranial devices, neurosurgical interventions and prevention of progression of intracranial hemorrhage. Outcomes associated with the receipt of blood products, including packed red blood cells (PRBC), plasma, platelets, cryoprecipitate, and/or whole blood in the setting of TBI are also worse [11]. Despite the association with adverse outcomes, limited pediatric studies have led to an absence of robust, evidence-based management principles in this field, as such the balance between the consequences of anemia and coagulopathy with adverse effects related to blood product transfusion has yet to be elucidated. In this review, we aimed to describe the pediatric literature on component and whole blood product transfusion in children with TBI and the pathophysiological mechanisms underlying the development of coagulopathy in this setting. We incorporated available pediatric TBI-specific guidelines and recommendations in our review.

## 2. Methods

We searched published literature for relevant articles from inception to present in the following databases: MEDLINE (Ovid), EMBASE (Elsevier), Web of Science or SCOPUS, Cochrane Library including CENTRAL and CDSR, clinicaltrials.gov, and WHO International Clinical Trials Registry Platform (ICTRP). We used the search terms PEDIATRICS, CHILDREN, TRAUMATIC BRAIN INJURY, BLOOD OR BLOOD TRANSFUSION. We manually reviewed the articles to determine eligibility for inclusion in our review. Reference lists of pertinent studies were also reviewed for relevant articles.

## 3. Definitions and Epidemiology of Coagulopathy and Transfusion in Pediatric TBI

The term acute traumatic coagulopathy (ATC) is an entity commonly described in trauma-related literature and refers to the endogenous coagulopathy that develops immediately after severe traumatic injury [12,13,14]. Mechanistically, it has been described to occur as a result of the combination of tissue injury and shock acquired from traumatic injury [15]. ATC is the initial process that precedes TIC, which encompasses ATC and iatrogenic coagulopathy resulting from therapeutic resuscitation, the latter referred to as “resuscitation-associated coagulopathy” (RAC) [15,16]. The classic “lethal triad” and more recent “lethal diamond” (including hypocalcemia) have been well-documented and simplistically describe these mechanisms [17]. However, it is essential to note that these definitions lack standardization and quantitative diagnostic criteria. Additionally, the underlying mechanisms involved are complex [4,18]. TBI-associated coagulopathy has been described in both adults and children and is thought to have pathways distinct from TIC [19,20]. It occurs in isolated TBI and does not generally follow widespread injury and/or hypoperfusion [21,22,23]. Similarities with disseminated intravascular coagulopathy (DIC) have been described, with some authors proposing that the mechanisms underlying DIC are similar, as the coagulopathy seen in patients with TBI consistently fits definitions proposed by the International Society on Thrombosis and Haemostasis (ISTH) [24,25]. Table 1 summarizes the various coagulopathy terminology described in trauma-related literature (Table 1).

The reported incidence of TIC in children is highly variable [5]. Standardization has been difficult primarily due to the absence of laboratory testing and specific thresholds to define TIC [7]. Multiple confounders exist in pediatric TBI literature due to its association with other traumatic injuries and an overall lack of studies examining isolated TBI alone in children. The reported incidence of coagulopathy specific to isolated TBI in children ranges anywhere from 15 to 87% [19]. The timeframe for the development of TBI-associated coagulopathy is also much debated. Both early and late temporal courses have been described, with some adult literature reporting its development within minutes after sTBI and others with a more protracted onset at 12 h to up to 4.5 days [26]. Blood transfusion rates in the setting of pediatric TBI, including all component blood products, are reported to be around 18.5% and 10–44% for PRBC alone [11]. Overall, due to the heterogeneity in studies and their populations, definitions, diagnostic thresholds, and proposed mechanisms, comparisons across studies have been difficult, and the characterization of the epidemiology of pediatric TBI has been challenging.

## 4. Pathophysiological Considerations and Concepts

### 4.1. Mechanisms Leading to TBI-Associated Coagulopathy

**Figure 1:**
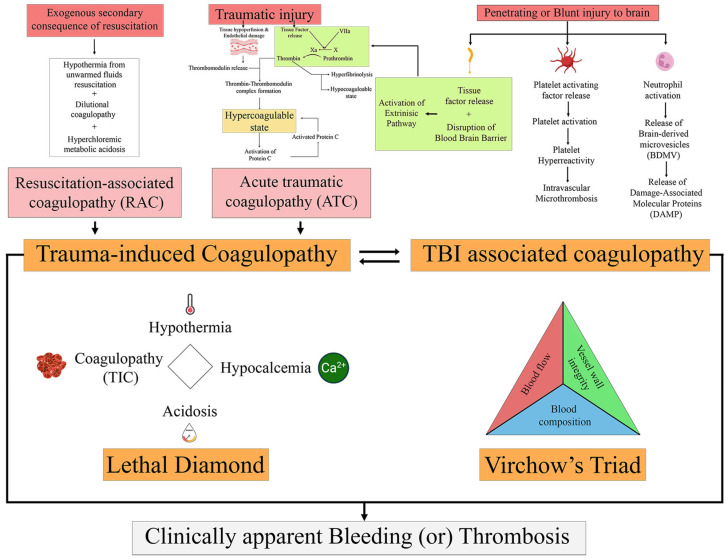
Various terminology used in trauma-related literature and their underlying mechanisms. TIC consists of endogenous and exogenous mechanisms, ATC and RAC, respectively. TBI-associated coagulopathy, similar to TIC, leads to tissue factor release with subsequent activation of the extrinsic pathway. Additionally, platelet and neutrophil activation are also thought to be play a role. The understanding and overlap between the link of the mechanisms involved, especially that between TIC and TBI-associated coagulopathy, continue to evolve. ATC—Acute traumatic coagulopathy; RAC—Resuscitation associated coagulopathy; TIC—Trauma-Induced Coagulopathy.

Brain injury is associated with the disruption of the coagulation and fibrinolytic system, leading to both coagulopathy and disordered fibrinolysis [27]. Patients with isolated TBI differ from those with extracranial injuries as they usually do not have massive blood loss and are less likely to receive large amounts of fluid resuscitation to avoid potentially worsening intracranial pressure. Thus, mechanisms underlying the development of TBI-associated coagulopathy are thought to be unique and independent of iatrogenic causes [9]. Various multifactorial mechanisms and cellular alterations have been proposed to explain the development of systemic hemostasis and coagulopathy from localized injury in the brain (Figure 1). A predominant hypothesis is that the central nervous system (CNS) is abundant in the membrane lipoprotein tissue factor, and that direct injury to the brain causes its release into the circulation via disruption of the endothelium and blood–brain barrier (BBB) [28]. Animal models have demonstrated this hypothesis, and human studies have documented the release of endothelium-specific biomarkers, including thrombomodulin and syndecan-1, after sTBI [29]. Overall, this leads to activation of the extrinsic coagulation pathway, including *activated protein C*, ultimately leading to thrombin generation with subsequent fibrin formation and hyperfibrinolysis. In the setting of other injuries, concomitant trauma-related hypoperfusion-induced dysregulation of the protein C system is also known to occur [30]. Secondary ischemic and inflammatory processes contribute to the process.

Activation of platelets leading to platelet hyperactivity is another identified pathway contributing to the development of coagulopathy, but the exact mechanisms remain unclear [8]. Multiple factors, including the release of platelet activating factor (PAF), one of the classic autacoids also found abundantly within the CNS, act via G-protein-coupled transmembrane receptors to activate circulating platelets, as suggested by animal studies and the presence of platelet aggregation and increased risk for intravascular microthrombosis in children with TBI [8]. Additionally, platelet-endothelial interactions, including those with von Willebrand factor and ADAMTS-13, and the contribution of phospholipids, including phosphatidylserine, are also likely involved, serving as a bridge between platelet activation and thrombin generation. Finally, inflammatory cells such as activated neutrophils and the neutrophil extracellular traps pathway, with subsequent release of other procoagulant factors, including brain-derived microvesicles and damage-associated molecular proteins, are also likely to be mechanistically involved. Overall, all these described mechanisms are only supported by circumstantial animal evidence and continue to evolve [8].

Ultimately, the release of tissue factor and other procoagulant factors, platelet activation, and endothelial interactions all likely contribute to the pathophysiology of TBI-associated coagulopathy, but direct evidence is lacking. A Japanese multicenter prospective study aimed to elucidate the mechanisms underlying TBI-associated coagulopathy and TIC and found that the coagulofibrinolytic changes in both were similar and consistent with those seen in DIC [24]. Both hypo- and hypercoagulability have been reported in pediatric TBI, with D-dimer and fibrinogen degradation products (FDP) detected within minutes of injury, followed by prolongation of PT and aPTT hours later, suggesting a consumptive pathophysiology [9]. Additionally, abnormal fibrinolysis and fibrinolytic shutdown are other phenotypes that occur in the setting of trauma and isolated TBI. It is thought to occur secondary to compensatory mechanisms, and its presence is associated with adverse events and poor outcomes [31,32].

### 4.2. TBI Management Principles and Considerations

The management of pediatric TBI is guided by the principles of cerebral oxygenation and cerebral perfusion pressure (CPP), with a goal to optimize both without causing cerebral edema. CPP is defined by mean arterial pressure minus the mean intracranial pressure (ICP) and reflects the pressure gradient that drives cerebral blood flow (CBF). It is regulated by autoregulation and the cerebral metabolic rate for oxygen. CPP management is often used to guide sTBI management [33]. Because cerebral hypoxia can occur despite adequate CPP, the use of brain tissue partial pressure of oxygen (PBrO_2_) monitoring has been more widely utilized in the management of severe TBI, with studies suggesting that higher PBrO_2_ (>10 mmHg) may be associated with better outcomes [34,35]. However, no studies have directly evaluated this. Children with TBI are thus likely to receive blood product transfusions to either improve oxygen delivery and/or correct coagulopathy. The concomitant presence of hemorrhage and shock of various etiology common in the setting of trauma (e.g., hemorrhagic shock, obstructive shock from tamponade physiology, neurogenic shock) is likely to affect the resuscitation of patients with TBI, and these patients are more likely to receive intravascular volume, including blood products.

The use of hyperosmolar therapy is mainstay in the management of increased ICP, which is the primary driver of mortality in children with sTBI [33]. Theoretically, the use of hyperosmolar therapy can potentially lead to adverse events in the setting of TBI, including dilutional coagulopathy, such as that seen with crystalloid resuscitation. However, randomized trials in adults have failed to show an association between these two [36,37]. The prolonged use of continuous hypertonic saline solution was found to be associated with increased rates of RBC and plasma transfusions [38]. Other potential complications related to hyperosmolar therapy in the setting of trauma and TBI are an increased risk for the development of hospital-acquired venous thromboembolism (HA-VTE) [39]. Guidelines for pediatric sTBI recommend keeping serum sodium levels below 160 mEq/L to avoid the complication of VTE. Similarly, these guidelines also suggest keeping serum sodium levels below 170 mEq/L to avoid the complications of anemia and thrombocytopenia. Overall, the effect of hyperosmolar therapy and its relation to TBI-associated coagulopathy and outcomes have not been well-studied. Trials in adults investigating the association of various hyperosmolar agents with the development of coagulopathy and inflammatory markers are currently ongoing [40].

Finally, the contribution of developmental hemostasis in pediatric TBI-associated coagulopathy also remains to be elucidated. Age-dependent differences and the effect of various pathologies on pediatric hemostatic balance are poorly understood mechanisms. In the setting of disruption of this balance via an accrual of risk factors, such as from inflamed injured states or the presence of foreign materials, e.g., central venous access devices, critically ill children afflicted with trauma with or without associated TBI are prone to not only bleeding, but also HA-VTE [41].

## 5. Blood Transfusion in the Setting of Pediatric TBI

### 5.1. Packed Red Blood Cells

PRBCs are the most utilized and transfused blood component regardless of clinical indication [42]. Hemorrhagic shock frequently occurs with TBI in the setting of polytrauma, confounding studies investigating the association between PRBC transfusion and outcomes in pediatric TBI alone. PRBC transfusion is thus utilized with a goal to improve oxygen delivery and correct symptoms related to anemia. However, oxygen delivery at the microcirculatory level is more complex than the Fick-derived oxygen delivery equation (cardiac output x arterial oxygen content), with the effect of viscosity on flow resistance (Poiseuille’s law) often not being fully considered [43,44]. Thus, the balance between anemia and increased viscosity in the setting of oxygen delivery remains poorly understood, not only in the realm of TBI, but in other transfusion-related situations as well. Additionally, there is a lack of literature regarding transfusion strategies targeting physiological parameters versus hemoglobin cut-offs. Thus, it remains unclear if PRBC transfusions can be tailored to individual physiological reserve. However, some adult studies using vascular occlusion and near-infrared spectroscopy suggest the potential for identification of populations who would benefit most from a PRBC transfusion [45].

In a small retrospective analysis, initial admission hemoglobin levels and averaged hemoglobin over 7 days in pediatric TBI were not associated with adverse outcomes [46]. Specific to sTBI in children, TBI guidelines suggest targeting a minimum hemoglobin goal of 7 g/dL, which was intentionally set to be consistent with current general PRBC transfusion recommendations for critically ill children with acute brain injury, considering transfusion to target hemoglobin levels between 7 and 10 g/dL [33,47] (Table 2). Current TBI guidelines for children do not support the use of invasive PBrO_2_ monitoring. However, if PBrO_2_ is utilized, a level greater than 10 mm Hg is recommended [33]. PBrO_2_ can be manipulated by increasing arterial oxygenation (increasing FiO_2_ and mean airway pressure, with the latter needing to be balanced with its potential effect on cerebral venous drainage), raising cardiac output with vasoactive substances, controlling minute ventilation to target PaCO_2_ levels to provide adequate CBF but avoid increases in ICP, and optimizing blood hemoglobin levels with PRBC transfusions [48]. However, the influence of PRBC transfusion on PBrO_2_ remains elusive, with studies demonstrating inconsistent improvement in local cerebral oxygenation and multiple limitations, including differences in response of damaged vs. undamaged brains and the effect of volume-related changes on cerebral perfusion itself [35]. Additionally, one of these studies found that the increased PBrO_2_ only occurred early after transfusion and with transient effects [35]. More importantly, the effect of increasing PBrO_2_ on clinical outcomes and its impact on cellular level oxygenation currently remains unclear.

Adults RCTs have had conflicting results when assessing the effect of a restrictive versus liberal transfusion strategy in TBI. A recent 2024 RCT in adults with acute brain injury found that those randomized to a liberal transfusion goal of less than 9 g/dL versus a restrictive goal of less than 7 g/dL were less likely to have unfavorable neurologic outcomes [51]. Contrary to this, another recent RCT in critically ill adults with TBI and anemia showed that a liberal transfusion strategy of PRBC transfusion given for a hemoglobin of 10 g/dL or lower did not show reduced risk of unfavorable neurologic outcome [52]. Most recent meta-analyses have concluded that there currently is no difference in unfavorable neurologic outcome or mortality despite controlling for Glasgow coma scale (GCS) scores between restrictive and liberal groups [53,54,55]. To date, no pediatric trials specific to PRBC transfusion and TBI exist.

### 5.2. Plasma and Its Components

The use of plasma and its components is typically used to address coagulopathy in various clinical settings by replenishing clotting factors. Plasma refers to the liquid component of blood containing both coagulation factors and inhibitors, and includes fresh frozen plasma (FFP), cryoprecipitate, thawed plasma, liquid plasma and cryoprecipitate poor plasma. Additionally, it is said to aid in the repair of endothelial injury and in mitigating excitotoxicity, thus protecting against cerebral edema and secondary brain injury [56]. Adult trials investigating the use of plasma, similar to that in PRBC trials, have had conflicting results [57,58,59,60,61]. However, recent studies investigating the use of prehospital resuscitation with plasma in severely injured patients have shown a mortality benefit, especially in those with sTBI [59,60,61]. Retrospective analysis of the National Trauma Databank in children with sTBI has shown that early plasma administration is associated with a lower risk of 4-h mortality, but overall associated mortality reported in studies remains high [62,63]. Moreso, prospective trials in pediatrics are lacking. Fibrinolytic shutdown after trauma is a more common occurrence seen in children compared to adults [64]. Prospective observational data in children have shown that delayed resuscitation with plasma in those already with established fibrinolytic shutdown has poor outcomes, and that over resuscitation with plasma in those that are not clinically coagulopathic or bleeding is independently associated with sustained fibrinolytic shutdown [49]. Overall, further studies are needed to investigate how the fibrinolytic system is affected in isolated pediatric TBI and the role of plasma therapy. There remains an absence of high-quality evidence to guide plasma transfusion strategies in critically ill children with TBI. Expert recommendations state that plasma transfusions for an INR ≤ 1.5 in the setting of placement of an ICP monitoring device may not be beneficial [50,65] (Table 2).

The literature on the use of cryoprecipitate in the setting of TBI is even more sparse, with no pediatric-specific literature available. Cryoprecipitate has the advantage of having higher concentrations of fibrinogen compared to fresh frozen plasma. A single-center retrospective cohort study in Tokyo, Japan, involving adults diagnosed with severe TBI and acute subdural hematoma who received empirical cryoprecipitate transfusion, found no adverse events. Due to multiple confounders, however, they concluded that cryoprecipitate might potentially lower rates of coagulopathy and mortality, but without strong certainty [66].

### 5.3. Platelets

Decreased platelet counts are also a frequent observation in children with TBI [67]. Prospective evaluation has suggested that the degree of decrease in platelet count is correlated to severity and prognosis of TBI, and that this is more relevant than the actual presence of thrombocytopenia [67,68,69]. Additionally, platelet dysfunction is also known to occur in moderate to severe TBI [70]. Platelet transfusions are thus utilized to treat or prevent bleeding regardless of the specific mechanism of TBI involved. In adult TBI patients, studies suggest specific initial platelet cut-offs associated with higher odds of intracranial hemorrhage progression, especially in those on prehospital antiplatelet therapy [68,69]. Prospective evaluation in adults receiving a massive transfusion protocol in the setting of trauma, not specific to isolated TBI, has documented a reduction in fibrinolysis but no improvement in clotting time, clot strength, platelet aggregation, and platelet count [71]. There has been no documented improvement in outcomes with platelet transfusions even in adult literature. As with plasma transfusions, no specific indications related to TBI have been defined in pediatrics based on current available literature. Most experts agree that platelet transfusions for platelet counts > 100,000/μL in this setting may not be beneficial and might have the potential for harm [11,50]. Platelet transfusions might be considered if an ICP monitoring device needs to be inserted in a neurologically deteriorating child with a platelet count < 100,000/μL [65,72] (Table 2).

### 5.4. Whole Blood

Pediatric literature on whole blood transfusion is mostly related to its use in the setting of injured children with hemorrhagic shock, with guidelines recommending its consideration for resuscitation [73,74]. In a propensity-matched cohort of children afflicted with trauma, including those with sTBI, those who received low-titer group O cold-stored whole blood had faster resolution of acidosis and received less plasma and platelet transfusion volume compared to component transfusion. Specific to pediatric TBI, few studies exist. Retrospective studies of the Trauma Quality Improvement Program database in 2020–2021 found that the use of whole blood in pediatric trauma patients with TBI had a lower risk of death in the first 24 h compared to those receiving component therapy [75]. Whole blood contains higher levels of nitric oxide and 2,3 diphosphoglycerate, has a higher hematocrit because of less hemodilution from additives, and has greater clotting factor activity [76]. Thus, the composition of whole blood theoretically induces less vasoconstriction and improves oxygen delivery, thereby improving cerebral oxygenation by increasing oxygen unloading to the tissues. Higher hematocrit theoretically may protect against cerebral edema. However, the effect of blood viscosity on cerebral flow mechanics and its interaction with the microcirculation, as previously mentioned, is complex.

## 6. Current State of Transfusion Practice and Knowledge Gaps

The absence of high-quality data constrains the current state of transfusion practice in pediatric TBI. Although outcomes associated with component blood product transfusions are poor, the drivers of these outcomes remain unclear. Efforts should be made to standardize definitions of TIC and TBI-associated coagulopathy in pediatrics and to include pediatric-specific cutoffs. Comparative pediatric trials specific to both isolated TBI and TBI in the setting of polytrauma that examine restrictive versus liberal hemoglobin targets, similar to those used in adults, are urgently needed. Studies examining transfusion indications and thresholds for plasma and platelets, including during invasive procedures such as ICP device placement, are essential. The use of whole blood versus component therapy for pediatric TBI warrants further investigation, as it may improve oxygen delivery and correct coagulopathy more efficiently than component therapy, thereby improving outcomes. The role of viscoelastic testing in pediatric TBI has not been addressed in this review, but pediatric transfusion guidelines suggest considering its use as an adjunct to standard coagulation tests. Finally, the roles of other hemostatic products, including antifibrinolytics such as tranexamic acid, factor VII concentrate, and prothrombin complex concentrate, have also not been covered in this review. However, they carry essential management implications that urgently need investigation.

## 7. Summary

Blood product transfusion is common in the setting of pediatric TBI and seems to be associated with poor outcomes. Although there is some adult evidence, the overall understanding of TBI-associated coagulopathy remains poor. Pathophysiological cellular mechanisms have been described, but direct investigations are lacking. Overall, the literature specific to pediatrics and isolated TBI remains sparse, limiting the ability to make robust recommendations to guide transfusions in these settings. Future studies in pediatric TBI are urgently needed, focusing on standardization of definitions, transfusion indications and thresholds, and the role of whole blood and other hemostatic products. In summary, we have reviewed literature specific to blood transfusions in pediatric TBI and found a paucity of specific data.

## Figures and Tables

**Table 1:** Definition of terms. Various coagulopathy related terminology used in trauma related literature.

Acute traumatic coagulopathy	Endogenous coagulopathy that develops after severe traumatic injury
Resuscitation-associated coagulopathy	Iatrogenic coagulopathy that results from therapeutic resuscitation
Trauma-induced coagulopathy	Combination of acute traumatic coagulopathy and resuscitation-associated coagulopathy
Disseminated intravascular coagulopathy	Acquired syndrome characterized by intravascular activation of coagulation, dysregulated fibrinolysis and endothelial injury with loss of localization, usually secondary to critical conditions
TBI-associated coagulopathy	Coagulopathy that occurs after isolated TBI or TBI with associated polytrauma

**Table 2:** Summary of existing recommendations for pediatric sTBI and blood product transfusions.

Recommendation	Rationale for Recommendations	Guideline/Expert Group	Type of Blood Product	Future Research Recommendations by Expert Groups
Hemoglobin target of at least 7 g/dL	Committee recommendations based on few published protocols. The lower threshold cut-off of 7 g/dL was chosen to be consistent with TAXI guidelines.	Supplemental clinical practice algorithm for sTBI management 2019 *	Packed RBC	No recommendations
Hemoglobin target of 7–10 g/dL	Strong consensus based on expert opinion. Based on sTBI protocols and adult systematic reviews to use 7 g/dL as lowest threshold and not transfuse when >10 g/dL.	TAXI 2018 **	Packed RBC	Future clinical trials testing transfusion threshold/hemoglobin concentration with best long-term functional outcomes. Further clinical physiology studies to evaluate role of PBrO_2_ monitoring.
General statement that treatment of abnormal coagulation variables is recommended prior to insertion of devices but with caution. No specific thresholds given.	Committee recommendations based on few published protocols and retrospective reviews. Caution on treatment of coagulopathy was recommended based on a prospective observational study suggesting over-resuscitation with plasma may worsen coagulopathy [49].	Supplement clinical practice algorithm for sTBI management 2019 *	Plasma	No recommendations
Plasma transfusion for INR ≤ 1.5 when placing an ICP device may not be beneficial	Strong consensus agreement based on expert opinion. Small observational studies noting no increased risk of hemorrhage when INR ≤ 1.5 during placement. No recommendations from other societies.	TAXI-CAB 2022 ***	Plasma	Future studies to determine if plasma should be administered for safety of device placement and at what laboratory threshold. General recommendation for future studies that describe laboratory thresholds examining risk/benefit of tolerating coagulation abnormality versus plasma transfusion.
Transfusion for >100,000/μL may not be beneficial and might be harmful in a neurologically stable child with moderate to severe TBI	Strong consensus agreement based on expert opinion. Insufficient evidence to guide specific threshold.	TAXI-CAB 2022 ***	Platelets	General recommendation for future studies that describe laboratory thresholds examining risk/benefit of tolerating thrombocytopenia versus platelet transfusion.
May be considered for <100,000/μL and ICP device needs to be inserted in a neurologically deteriorating child	Strong consensus agreement based on expert opinion. No current studies addressing this. No recommendations from other societies.	TAXI-CAB 2022 ***	Platelets	Future studies to determine if platelets should be administered for safety of device placement and at what laboratory threshold.

*—Management of Pediatric Severe Traumatic Brain Injury: 2019 Consensus and Guidelines-Based Algorithm for First and Second Tier Therapies by Kochanek et al. 2019 [33]. **—Consensus Recommendations for RBC Transfusion Practice in Critically Ill Children From the Pediatric Critical Care Transfusion and Anemia Expertise Initiative (TAXI) by Valentine et al. 2018 [47]. ***—Executive Summary of Recommendations and Expert Consensus for Plasma and Platelet Transfusion Practice in Critically Ill Children: From the Transfusion and Anemia EXpertise Initiative-Control/Avoidance of Bleeding (TAXI-CAB) by Nellis et al. 2022 [50].

## Data Availability

No new data were created or analyzed in this study. Data sharing is not applicable to this article.
